# Dehydration and Malnutrition in Residential Care: Recommendations for Strategies for Improving Practice Derived from a Scoping Review of Existing Policies and Guidelines

**DOI:** 10.3390/geriatrics3040077

**Published:** 2018-11-12

**Authors:** Diane Bunn, Lee Hooper, Ailsa Welch

**Affiliations:** 1 School of Health Sciences, Norwich Research Park, University of East Anglia, Norwich NR4 7TJ, UK; 2 Norwich Medical School, Norwich Research Park, University of East Anglia, Norwich NR4 7TJ, UK; l.hooper@uea.ac.uk (L.H.); a.welch@uea.ac.uk (A.W.)

**Keywords:** aged, homes for the aged, malnutrition, dehydration, nutrition policy

## Abstract

Preventing malnutrition and dehydration in older care home residents is a complex task, with both conditions remaining prevalent, despite numerous guidelines spanning several decades. This policy-mapping scoping review used snowballing search methods to locate publicly-available policies, reports and best practice guidelines relating to hydration and nutrition in UK residential care homes, to describe the existing knowledge base and pinpoint gaps in practice, interpretation and further investigation. The findings were synthesised narratively to identify solutions. Strategies for improvements to nutritional and hydration care include the development of age and population-specific nutrient and fluid intake guidelines, statutory regulation, contractual obligations for commissioners, appropriate menu-planning, the implementation and auditing of care, acknowledgment of residents’ eating and drinking experiences, effective screening, monitoring and treatment and staff training. The considerable body of existing knowledge is failing to influence practice, relating to translational issues of implementing knowledge into care at the point of delivery, and this is where future research and actions should focus.

## 1. Introduction

There is widespread consensus that malnutrition and dehydration are prevalent conditions in older people living in residential care, and have been so for many years, as evidenced by numerous research papers and publications by governments, charitable and professional bodies. Many of these documents include recommendations for combatting these largely preventable conditions. Even so, malnutrition and dehydration in care homes remain ongoing issues, which may be indicative of the complexities associated with providing effective nutritional and hydration care for this population [1,2,3,4,5].

Over the last three years, at least another ten published reports have highlighted aspects of nutrition, hydration and supplementation in UK care homes:

- Between 2014–2017, the Care Quality Commission (CQC) took approximately 50 enforcement actions against care homes for breaching nutrition and hydration regulations [6].
- A UK consensus study to establish research priorities in care homes identified nutritional and hydration care as essential due tothe poor nutritional status of many care home residents [7].
- 20% of older people living in UK care homes were dehydrated [8].
- The National Health Service (NHS) framework for enhanced health in care homes recommended nutrition and hydration support as core requirements [9].
- The Scientific Advisory Committee on Nutrition (SACN) [10], the National Institute of Health and Clinical Excellence (NICE) [11,12], Health Improvement Scotland [13] and the National Osteoporosis Society [14] each recommended vitamin D supplementation in all older people to improve skeletal health.
- The British Association for Parenteral and Enteral Nutrition (BAPEN) reported 34% of older care home residents were malnourished [15].
- The British Dietetic Association issued a policy statement highlighting the growing issue of malnutrition in older people living in care homes, and the dietitian’s role in prevention [16].

These findings and recommendations are not new, they are just the most recent publications representing a plethora of reports attempting to address this crucial area, and this is not only limited to the UK (see for example References [17,18,19,20,21]).

Malnutrition is defined as a deficiency of one or more nutrients resulting in measurable adverse effects on body composition, function or clinical outcome [22] whilst low-intake dehydration is defined as a deficiency of water due to insufficient drinking [23]. Malnutrition is a risk factor for sarcopenia and frailty [21,24,25], and both conditions increase vulnerability to adverse outcomes and limit quality of life, health and well-being [26,27,28]. An estimated 90% of older care home residents have osteoporosis and one third are vitamin D deficient [14,19,29,30]. Blood vitamin C concentrations indicative of scurvy are present in 40% of residents [4]. These are likely related to poor nutrition and in the case of osteoporosis, a lack of vitamin D supplementation. Other micronutrient deficiencies are also highly prevalent [4]. Despite NICE recommendations for vitamin D supplementation in at-risk groups in the UK, our recent study [8] found just 23% of 200 older adults aged 65–104 years living in residential care were prescribed vitamin D (DRIE, Dehydration Recognition in our Elders study, unpublished data), and this has been confirmed by other UK studies [31]. Low-intake dehydration is found in 20% of older adults living in residential care and is associated with an increased risk of disability, falls, infections, unplanned hospital admission and mortality [28,32]. 

Looking forward, increased life expectancy and increasing numbers of people reaching older ages means that more older people will require residential care [33,34,35]. Effectively addressing malnutrition and dehydration in older care home residents is urgently required, both to improve health and quality of life, and to reduce the burden on healthcare systems [36,37]. However, knowledge of malnutrition and dehydration prevalence is failing to influence practice. 

The aim of this scoping review was to identify and map publicly-accessible policies, guidelines and “best practice” documents relating to hydration, nutrition and nutritional supplementation in UK residential care homes to identify the key components of effective nutritional and hydration care, describe the existing knowledge base and identify gaps in existing literature to signpost areas of future investigation and research.

## 2. Materials and Methods

We followed the framework of Arksey and O’Malley [38] to identify policies, reports and best-practice guidelines for this policy-mapping scoping review, and using snowballing search methods, as these documents are not indexed by standard bibliographic databases [39,40]. Key documents were initially identified by the research team and one reviewer (DB) checked the cited references, using judgement to determine which ones to pursue further, as well as forward-tracking relevant cited documents. In addition, we used our professional networks to alert us to relevant documents published over the course of the study. We included documents relating to nutrition, hydration and/or nutritional supplementation in UK care home settings specifically, and in older people generally, if the setting included older people living in care homes (Supplementary Table S1). We tabulated key points and undertook a narrative synthesis to identify key components of effective nutrition and hydration care in care homes, and to describe the extent of existing knowledge with a focus on identifying areas for future research.

As a scoping review of publicly-available documents, ethical approval was not required for this study. 

## 3. Results

We retrieved 79 documents, 75 of which were from the UK, but we included one published by the Council of Europe and three published by WHO, as both organisations may influence UK policy. Of the 75 UK documents, 60 were published by public bodies (including NICE, SACN, Public Health England, Food Standards Agency), nine by charitable organisations (Age UK, BAPEN, Caroline Walker Trust) and six by professional bodies (such as the British Dietetic Association). All were broadly relevant to older people living in residential care settings, but 28 of these related specifically to older people living in residential care.

Narrative synthesis identified eight key aspects of nutritional and hydration care in care homes that need to be addressed. These are outlined in Table 1 and discussed in detail below.

### 3.1. Focussed Regulation of Nutritional and Hydration Care

The UK independent health and social care regulators are the CQC (England and Wales) [41], the Care Inspectorate (Scotland) [42] and the Regulation and Quality Improvement Authority (Northern Ireland) [43,44]. For all three regulators, nutrition and hydration in care homes are key components of their regulatory duties, but the regulations only provide broad outlines as to what nutrition and hydration care should entail, rather than specifically detailing how they should be achieved [42,45,46,47,48,49,50,51,52,53,54,55]. Alongside these regulations, government agencies in all four UK countries have each published a number of standards and guidelines covering nutrition and hydration care in care homes in various detail, but it is unclear how these guidelines should map to the regulator’s requirements to fulfil the requirements of the regulations. We suggest that the implementation of existing guidelines is integrated into existing regulatory systems.

### 3.2. Commissioning Nutritional and Hydration Care

Care home ownership falls into three broad categories: Private sector (owned by individuals, partnerships, public and private limited companies), voluntary sector (owned by charities) and the public sector (owned and managed by local authorities or NHS trusts). Apart from those owned and managed by NHS trusts, care homes come under the auspices of Local Authority Social Care [56].

Local authorities, as well as the NHS, are major commissioners of services. As commissioners, the NHS and local authorities can be influential in improving hydration and nutritional care in care homes, by ensuring that nutrition and hydration care are in the contracting, quality assurance and performance monitoring of commissioned services [57,58]. We agree with NHS England’s conclusions that the commissioning process should take an integrated approach, acknowledging the psychological, physical and social aspects of nutrition and hydration, but as with regulation, further guidance is needed to map existing nutrition and hydration guidelines to appropriate policies for commissioning services. 

### 3.3. Nutrient, Dietary and Supplement Guidelines for Older People >65 Years

Nutrient and dietary guidelines have been published by several UK organisations [10,11,12,13,46,54,59,60,61,62,63,64,65,66,67,68,69,70,71,72,73,74], but most track back to the 1991 Committee on Medical Aspects of Food Policy (COMA) Report [63], although SACN has recently updated findings on certain nutrients, including vitamin D [10].

Beyond the UK, the European Council [75] and World Health Organization (WHO) [76] have each published documents relating to nutritional care for older people, although only the European Council document refers specifically to older people living in care homes. In addition, both the European Food Safety Agency and the WHO have produced guidelines relating to specific nutrients, some of which may be applicable to older people living in care homes, but they have not developed evidence-based guidelines for older residents with respect to their particular problems and requirements [77].

### 3.4. Menu Planning and Catering

Catering involves the procurement and sourcing of foods, food safety, food waste and related ethical principles, whereas menu planning addresses how menus are planned to meet nutrient and dietary guidelines for care home residents. Several UK reports address both these issues [46,54,59,60,61,66,67,68,78], but there is no statutory requirement for care homes to follow these guidelines. We recommend that detailed guidance on the implementation of these guidelines is included in the regulatory process.

### 3.5. Residents’ Eating and Drinking Experience

Care home residents’ eating and drinking experiences are crucial aspects of nutritional and hydration care and should be fully acknowledged when addressing malnutrition and dehydration. Key aspects include [46,54,60,61,68,79,80,81,82,83]:

- Residents’ eating and drinking skills.
- Available eating and drinking assistance.
- Oral health.
- Swallowing abilities.
- Sensory abilities.
- Appetite and anorexia of aging.
- Exercise.
- Individual, cultural and/or religious preferences.
- Dietary needs.
- Food presentation.
- Social and physical environment.
- Institutional systems and organisation.
- Support from health professionals, including dietitians, and speech and language therapists.

Historically, many reports discussing residents’ experiences have failed to involve the residents themselves. Although more recent work increasingly includes residents’ and carers’ perspectives, more resident involvement is needed to identify outcomes in care home practice and research [84].

### 3.6. Screening and Monitoring

Screening identifies people at risk of malnutrition and dehydration with a view for instigating an intervention; whereas monitoring is the ongoing assessment of nutritional and hydration status. The recent Global Leadership Initiative on Malnutrition recommended that malnutrition risk screening should be undertaken on anyone coming into contact with health professionals [18].

In the UK, the most commonly-used nutrition screening tools are the Malnutrition Universal Screening Tool (MUST) [85], and the Mini Nutritional Assessment (MNA) [86]. Key documents addressing screening and monitoring in care homes [15,54,60,61,79,87] include a BAPEN report summarising the results of four surveys undertaken in 2007–2011, finding that malnutrition was present in 34% of residents, despite most care homes using MUST and having a nutritional screening policy. Using recognised nutritional screening tools is not compulsory [88], but is considered good practice, despite the lack of supporting evidence demonstrating how residents identified as being at risk of malnutrition are subsequently treated, or whether assessing nutritional status predicts outcome [89].

There is currently no effective screening tool for dehydration validated in older people, aside from the reference standard; serum or plasma osmolality [83,90,91]. To address this deficit, some organisations have developed their own risk assessment tools [92], attempting to identify those at most risk. However, without validation, it is not known whether the tools work, with the possibility that the reliance on a misleading tool is harmful.

There is a need to develop and share care pathways that include the identification of malnutrition and dehydration risk accompanied by guidance regarding subsequent interventions, as in the USA, where the Resident Assessment Instrument (a guide for identifying problems and treatment pathways) is mandatory in all care homes [93].

### 3.7. Implementing and Auditing Change

Implementing nutritional guidelines is key, as addressed by several reports [47,48,49,52,54,87,94,95,96,97,98], but details are often scanty on how the implementation was achieved and sustained, in order to enable the replication of best practice. Studies investigating the sustainability of interventions post-withdrawal of the research team report mixed findings, and a recent systematic review investigating interventions to change care home staff practices concluded that this was complex and challenging [99]. Recent revealing research described three barriers to implementing the Food Standards Agency’s nutritional guidelines in care homes [97]:

- Some staff felt that guidelines were irrelevant, and were seen as being restrictive.
- For staff who could see the relevance of guidelines, implementation was hampered by a lack of nutritional knowledge and institutional support.
- Staff perceived that residents in their care were not benefitting from the guidelines.

Developing a national compulsory nutritional audit tool may support the implementation of a cohesive nutritional and hydration care pathway [100].

### 3.8. Training in Nutritional and Hydration Care

CQC guidelines stipulate that nutrition and hydration assessments should be conducted by staff who have the necessary skills and knowledge, and training enables staff to acquire the ability to deliver appropriate nutritional care [79,101]. However, what essential knowledge is required and what training is needed to provide this is unclear. There is no formal requirement or agreed standards for care home staff regarding nutritional and hydration care [102] so training is often ‘ad hoc’, poorly supported and under-funded.

The Association of Nutrition identified training needs and competencies for those delivering nutritional care in care homes at different levels [103], starting at levels 3/4 (equivalent to the ‘A’ level standard). We would recommend a wider range of training opportunities be developed that are applicable to all care staff roles.

Nurses working in care homes are expected to adhere to the Nursing and Midwifery (NMC) Code of Practice, where hydration and nutrition are explicitly listed as one of the six fundamental aspects of care, thus requiring nurses to be knowledgeable about appropriate care.

## 4. Discussion

We have identified that effective nutritional and hydration care pathways in care homes should encompass eight key areas, as summarised in Table 1. There are a number of guidelines, policies and best practice documents addressing regulation, commissioning, dietary guidelines, vitamin D supplementation, menu planning and catering issues, but there are major gaps in how this considerable body of existing knowledge is failing to influence practice. This is due to translational issues of implementation to improve care at the point of delivery. Good nutrition and hydration is essential for resident well-being, and is a fundamental aspect of nursing care [104,105]. Further research is needed to provide evidence to guide nursing interventions [80,81,83,106]. 

This is the first scoping review which we are aware of that focusses on UK residential care, aiming to map policies and guidelines influencing nutrition and hydration care, and to provide an in-depth analysis. Scoping reviews, whilst recognised as being non-systematic, are valued by commissioners and policymakers as useful tools to identify and map wider literature, much of which is not published in peer-reviewed journals, such that questions and topics for future research and service development can be identified [40]. The snowballing search techniques utilised in this review were wide-ranging and used in the knowledge synthesis, developing the eight components of an effective nutritional care pathway. In our knowledge synthesis we reached saturation point, where ongoing synthesis reinforced our findings with no new components being identified.

In recognising that malnutrition and dehydration are prevalent in the UK, numerous agencies have found resources to comment on the current state and make suggestions as to how to improve, and there are good examples of successful initiatives, but these initiatives need to be more widespread, with supporting evidence of their sustainability.

Whilst we have described the situation in detail in the UK, malnutrition and dehydration are prevalent in many other countries worldwide [20], including Europe [17,75], North America [107,108] and Australia [109,110], thus the conclusions we have drawn are likely to be applicable internationally, despite differences in healthcare systems and guiding policies between individual countries.

Further research is needed to understand how to successfully translate knowledge into care at the point of delivery. The development of guidelines for resolving these issues is urgently needed.

## 5. Conclusions

Malnutrition, dehydration and low rates of vitamin D supplementation remain prevalent in older care home residents, and are associated with poor quality of life, comorbidities, increased hospital admissions and mortality. 

Effective nutritional and hydration care pathways in care homes should encompass eight key areas (Table 1), and whilst there are many documents addressing regulation, commissioning, dietary guidelines, vitamin D supplementation, menu planning and catering issues, there are major gaps in how these documents influence practice. Further work is required to identify how to pull together the considerable body of documents into a comprehensive set of readily accessible guidelines to address implementation, including the residents’ eating and drinking experience, screening and monitoring, staff training, implementing and auditing change. The authors and several organisations, including the National Osteoporosis Society, are working to develop solutions to these issues of implementation of guidelines for nutrition and hydration in care homes.

## Figures and Tables

**Table 1:** Key aspects of nutritional and hydration care in care homes.

Aspect	Summary of Findings	Summary of Recommendations
(i) Regulation	There are government guidelines and regulations in place, but they lack detail about how recommendations should be implemented.	To ensure existing guidelines are implemented appropriately, they should be integrated into the existing regulatory systems.
(ii) Commissioning	Commissioners of services can influence nutritional and hydration care in care homes.	Guidance is needed to map existing nutrition and hydration guidelines to appropriate policies for commissioning of services.
(iii) Dietary guidelines	There are a considerable number of nutritional and supplementation guidelines in place in the UK.	Age and context-specific guidelines relating to specific nutrients, should be developed for people aged >65 years living in care homes.
(iv) Menu planning and catering issues	There are a considerable number of reports and guidelines in place regarding catering and menu planning in care homes.	Implementation of existing guidelines should be a constituent of the regulatory process.
(v) Residents’ eating and drinking experience	The resident’s eating and drinking experience is crucial in preventing malnutrition and dehydration, and whilst a number of reports recognize this, further insights from residents themselves may highlight other factors.	Further research and increased involvement of residents, families and care staff is required to identify positive and negative practices associated with nutrition and hydration care in care homes.
(vi) Screening and monitoring	Screening and monitoring are recognised as important aspects of nutritional care when used appropriately. There is currently no validated tool assessing dehydration risk.	Clear care pathways to be identified where screening for malnutrition and dehydration risk includes specific courses of action for appropriate care.
(vii) Implementing and auditing change	Implementing guidelines for nutritional care is crucial for preventing malnutrition and dehydration, but effecting permanent change requires a structured approach involving all staff. A national audit tool may aid implementation.	The development of a national compulsory nutritional audit tool to support the implementation of a cohesive nutritional care pathway.
(viii) Staff training	Staff providing nutrition and hydration care in care homes should have the necessary skills and knowledge, but the details of what these skills and knowledge should be has not been defined.	Nationwide training competencies to be developed that are applicable to all care home staff roles.

## Supplementary Materials

The following are available online at http://www.mdpi.com/2308-3417/3/4/77/s1. Table S1: UK publicly-available policies, reports and best practice guidelines regarding Nutrition and Hydration care in care homes.
