# Supplementary material for: Dehydration and Malnutrition in Residential Care: Recommendations for Strategies for Improving Practice Derived from a Scoping Review of Existing Policies and Guidelines
Source: Geriatrics (Basel). 2018 Nov 12;3(4):77. doi: 10.3390/geriatrics3040077 (PMC6371146; doi:10.3390/geriatrics3040077)
Supplement: Supplementary file 1 [file geriatrics-03-00077-s001.pdf]

Supplementary Table 1: UK publicly-available policies, reports and best practice guidelines regarding Nutrition and Hydration care in care homes

Funding type	Organisation	Title	Date	Setting	Summary
Charity	Age UK (West Cumbria)	Target: Well-being Nutrition Scoping	2009	care homes	Nutrition Scoping exercise in Northwest Cumbria Community Research Service to find the most recent and relevant documents concerning nutrition-related practice for older people in care homes.
Charity	Age UK	My Home Life, Issue 18	2016	care homes	Mealtimes are a big part of everyone's day and food is a subject everyone loves to talk about. Eating and drinking well is an important part of keeping healthy and happy. The way food is offered, how and where it is served and the choices that are given make a real difference. Sometimes the smallest things have the biggest impact. This bulletin captured lots of ideas from care homes around the UK to share.<br>Website: www.ageuk.org.uk.
Charity	BAPEN (British association for parenteral and enteral nutrition)	Nutrition Screening Surveys in Care Homes in England	2015	care homes	Summarises findings of 4 surveys of UK hospitals, care homes and mental health units between 2007 and 2011. The surveys aimed to establish the prevalence of 'malnutrition' in the different care settings, to document current screening practice and identify problems that needed addressing and to provide feedback to local centres. This report is based on the amalgamated data from the UK care homes that participated in the four surveys, providing new information on the trends in nutritional care over time.<br>Website: https://www.bapen.org.uk.

Funding type	Organisation	Title	Date	Setting	Summary
Academic	Bournemouth University on behalf of the Burdett Trust for Nursing	Empowering Nurses and Care Home Staff to Lead Excellence in Nutrition and Dignity In Dementia Care Through Evidence Based Learning to Enhance the Care Environment. Final report	2016	care homes: dementia-specific	The overall aim of the project was to identify best practice for delivering nutrition in dementia care by providing a new nutrition education programme based on fundamental principles of self-leadership and nutrition to empower nurses and care home staff. Website: https://research.bournemouth.ac.uk/2017/05/eating-and-drinking-well-supporting-people-living-with-dementia/
UK Public body	Care Quality Commission (CQC)	Time to listen In care homes Dignity and nutrition inspection programme	2012	care homes	Looked at dignity and nutrition issues for older people living in 500 care homes across England, covering both homes that provide nursing care and those that do not. Website: http://www.cqc.org.uk/sites/default/files/documents/time_to_listen_-_care_homes_main_report_tag.pdf
UK Public body	CQC	Health and social care act Regulation 14: Meeting nutritional and hydration needs		care homes	CQC regulation ensuring that people who use services have adequate nutrition and hydration to sustain life and good health and reduce the risks of malnutrition and dehydration while they receive care and treatment. Website: https://www.cqc.org.uk/.
UK public body	CQC	The State of Adult Social Care Services	2017	care homes	Reports on what the CQC found about the quality of care across the whole range of adult social care services that they regulate. Website: https://www.cqc.org.uk/.

Funding type	Organisation	Title	Date	Setting	Summary
Public body	Council of Europe	Nutrition in care homes and home care - From recommendations to action (2009)	2009	care homes	Reporting on the prevalence, causes and specific consequences of undernutrition in care homes and home care.<br>Website: http://book.coe.int
UK public body	Derbyshire County Council	Care homes: a literature Review	2013	care homes	This literature review aimed to expand on the findings of the previous literature review, with the following objectives: to report the prevalence of diseases amongst care home residents, and identify any conditions where prevalence is higher amongst care home residents compared to the elderly population residing in the community compare the rates of hospital admissions amongst care home residents with the elderly population living in the community, and identify the main diagnoses that result in admissions for care home residents identify interventions that are effective at reducing hospital admissions among care home residents.<br>Website: https://observatory.derbyshire.gov.uk/.
UK Public body	Department of Health (DOH)	Care Homes for Older People: National Minimum Standards Care Homes Regulations 3rd Edition 2000	2003	care homes	A statement of national minimum standards of care homes covering all aspects of care. Published by the Secretary of State for Health under section 23(1) of the Care Standards Act 2000. Meals & nutrition covered on p.13-16.<br>Website:<br>http://www.dignityincare.org.uk/_library/resources/dignity/csipcomment/csci_national_minimum_standards.pdf
UK Public body	Food standards agency (FSA)	Example menus for care homes	2007	care homes	To develop a one-week example menu plan, menu 1, with recipes and full nutrient analysis for older people, over 75 years, in care homes, to assist care caterers in menu planning.<br>A further one-week menu cycle, menu 2, is also included.<br>Website: http://www.food.gov.uk/sites/default/files/multimedia/pdfs/carehomemenus.pdf
UK Public body	Food standards agency	Guidance on food served to older people in residential care	2007	care homes	This document provides specific guidance to care homes for older people who do not have nutritional requirements due to illness or disease.<br>Website: http://www.food.gov.uk/sites/default/files/multimedia/pdfs/olderresident.pdf

Funding type	Organisation	Title	Date	Setting	Summary
UK Public body	Malnutrition Task Force (DOH-funded)	Malnutrition in Later Life: Prevention and Early Intervention Best Practice Principles & Implementation Guide (Care Homes)	2013	Care homes	This guide defines the principles of best practice, the moral, legal, quality and financial case for change and practical advice to support a wide range of health, social care and voluntary organisations in making the required changes to counter malnutrition.<br>Website: http://www.malnutritiontaskforce.org.uk/wp-content/uploads/2014/07/Prevention_Early_Intervention_Of_Malnutrition_in_Later_Life_Care_Home.pdf
UK Public body	National Institute of Clinical Excellence (NICE)	Older people in care homes.	2015	care homes	This briefing summarises NICE's key recommendations for local authorities and partner organisations on the health and care of older people in care homes. It also highlights relevant quality standards. One paragraph on malnutrition - saying it needs to be addressed.<br>Website: http://www.nice.org.uk/advice/lgb25/resources/older-people-in-care-homes-60521208572869
UK public body	NICE	Oral health in care homes. Quality standard.	2017	older people, care homes	Adults who move into a care home have their mouth care needs assessed on admission.<br>Website: nice.org.uk/guidance/qs151
UK Public body	Northern Ireland (NI), Public Health Agency	Nutritional guidelines and menu checklist	2014	care homes	Aims of the guidelines:<br>• To encourage and support the provision of a balanced diet to individuals in residential and nursing homes in Northern Ireland.<br>• To provide additional information on older adults' dietary needs and related nutrition disorders.<br>• To highlight the importance of identifying and addressing malnutrition.<br>• To provide practical guidance and tools for menu planning and modifying food and drinks.<br>Website:<br>http://www.publichealth.hscni.net/sites/default/files/Nutritional_guidlines_and_menu_checklist_march_2014.pdf

Funding type	Organisation	Title	Date	Setting	Summary
UK Public body	NI Government (The Regulation & Quality Improvement Authority)	Promoting Good Nutrition<br>A Strategy for good nutritional care for adults in all care settings in Northern Ireland 2011-2016	2011	care homes	A Strategy for Good Nutritional Care for Adults in all Care Settings in Northern Ireland, the 10 Key Characteristics of the Council of Europe Resolution on Food and Nutritional Care in Health and Social Care Settings sets the scene for the development of a framework for action, by describing what good nutritional care looks like for each characteristic.<br>Website; https://www.health-ni.gov.uk/sites/default/files/publications/dhssps/promoting-good-nutrition_0.pdf
UK Public body	Public Health England	Healthier and More Sustainable Catering<br>A toolkit for serving food to older people in residential care	2014	care homes	This toolkit contains practical information and useful tips to help those working within residential care settings to buy, cook and serve healthier, more sustainable food.<br>Sections:<br>4.2 This toolkit also provides examples of analysed menus that meet the target recommendations described in Healthier and More Sustainable Catering: Nutrition Principles.<br>4.3 Information on food hygiene, cultural/religious needs, allergy and calorie labelling is also covered.<br>4.4 Practical information included in this toolkit refers to Government Buying Standards for Food and Catering Services (GBSF, see Appendix 1). GBSF ensures that:<br>- foods procured are served to higher nutritional standards<br>- foods are produced to higher sustainability standards and<br>- procurement of catering operations meets higher sustainability standards eg through reducing food waste.<br>Website:<br>https://www.gov.uk/government/uploads/system/uploads/attachment_data/file/347890/Older_people_toolkit.pdf

Funding type	Organisation	Title	Date	Setting	Summary
UK public body	Public Health England	Healthier and More Sustainable Catering: A toolkit for serving food to older people in residential care	2017	care homes	This toolkit contains practical information and useful tips to help those working within residential care settings to buy, cook and serve healthier, more sustainable food.<br>Sections:<br>4.2 Information on food hygiene, cultural/religious needs, allergy and calorie labelling is also covered.<br>4.3 Practical information included in this toolkit refers to Government Buying Standards for Food and Catering Services (see Appendix 1). GBSF ensures that:<br>• foods procured are served to higher nutritional standards<br>• foods are produced to higher sustainability standards and<br>• procurement of catering operations meets higher sustainability standards eg through reducing food waste<br>4.4 The Food Standards Agency provides advice on good food hygiene in Appendix 3, including how to handle foods that need extra care, cleaning, cooking and food storage; advice on allergens is also available at Appendix 4.<br>Website: https://assets.publishing.service.gov.uk/
UK Public body	Scotland, The Care Commission	Promoting nutrition in care homes for older people	2009	care homes	This report evaluates a programme aimed at improving nutrition in Scotland's care homes for older people.
UK Public body	Scotland, The Care Commission	Eating well in care homes for older people	2009	care homes	Information about the policies and procedures which care services should have in place. Recommendations provided about what all care homes should do.<br>Website: http://www.holyroodpr.co.uk/images/uploads/documents/18SEPeatingwell.pdf
UK Public body	Scotland, Centre for Health & Social Research	The Nutrition of elderly people and nutritional aspects of their care in long-term settings	2000	care homes	Audit report. A national audit to identify nutritional problems and improve nutrition in older care home residents.<br>Website:<br>http://admin.1fife.org.uk/weborgs/nhs/uploadfiles/publications/c64_CHSR_Nutrition_Residential_Care_Older_People.pdf

Funding type	Organisation	Title	Date	Setting	Summary
UK Public body	Social Care and Social Work Improvement Scotland (SCSWIS).	National care standards for care homes for older people	2007	care homes	Re care standards in general. Nutrition mentioned, but no detail provided.<br>Website: http://www.gov.scot/Resource/Doc/349525/0116836.pdf
**UK Public body**	Social Care Institute for Excellence (SCIE)	Nutritional care and hydration	2009	care homes	This Dignity in Care guide aims to:<br>• examine why the problem of malnutrition in older people persists, despite a raft of policy and guidance<br>• offer some solutions to the various problems that prevent change from happening<br>• highlight the key messages from guidance on nutrition (see Practice points)<br>• ensure that guidance is accessible to the social care sector by providing links to key documents (see Resources).
UK Public body	SCIE	Nutritional care and older people	2009	care homes	This 'At a Glance' summary examines nutritional care in relation to older people. As part of its Nutrition Action Plan, the Department of Health has asked SCIE to provide guidance on nutrition for the social care sector. SCIE's Dignity in Care guide has been expanded to cover this information in a section on Nutritional care.<br>Website: http://www.scie.org.uk/publications/ataglance/ataglance03.pdf
UK Public body	SCIE	Commissioning care homes: common safeguarding challenges	2012	care homes	Nutrition is a section and it refers to the 'at a glance' document above (http://www.scie.org.uk/publications/ataglance/ataglance03.pdf).
UK Public body	Social Care Institute for excellence (SCIE)	Dignity in care: Eating & nutritional care	current	care homes	This is from the SCIE website (https://www.scie.org.uk/) which covers a number of care issues relating to dignity (eg abuse, personal care).<br>Website covers screening, and practical tips to aid eating and drinking, rather than nutritional content of food.

Funding type	Organisation	Title	Date	Setting	Summary
UK Public Body	Wessex Academic Health Science Network (AHSN)	The Nourish Resource Pack	2017	care homes	Resource to promote nutrition and hydration in care homes.<br>Website: http://wessexahsn.org.uk/img/projects/Nourish%20Resource%20Pack%20Poster.pdf
